# Total Dietary Antioxidant Capacity and Longitudinal Trajectories of Body Composition

**DOI:** 10.3390/antiox9080728

**Published:** 2020-08-10

**Authors:** Niels van der Schaft, Katerina Trajanoska, Fernando Rivadeneira, M. Arfan Ikram, Josje D. Schoufour, Trudy Voortman

**Affiliations:** 1 Department of Epidemiology, Erasmus University Medical Center, 3015 GD Rotterdam, The Netherlands; n.vanderschaft@erasmusmc.nl (N.v.d.S.); k.trajanoska@erasmusmc.nl (K.T.); f.rivadeneira@erasmusmc.nl (F.R.); m.a.ikram@erasmusmc.nl (M.A.I.); j.d.schoufour@hva.nl (J.D.S.); 2 Department of Internal Medicine, Erasmus University Medical Center, 3015 GD Rotterdam, The Netherlands; 3 Faculty of Sports and Nutrition/Faculty of Health, center of Expertise Urban Vitality, Amsterdam University of Applied Sciences, 1097 DZ Amsterdam, The Netherlands

**Keywords:** dietary antioxidants, nutrition, body composition, diet, dietary antioxidant capacity, muscle strength

## Abstract

Although there is some evidence that total dietary antioxidant capacity (TDAC) is inversely associated with the presence of obesity, no longitudinal studies have been performed investigating the effect of TDAC on comprehensive measures of body composition over time. In this study, we included 4595 middle-aged and elderly participants from the Rotterdam Study, a population-based cohort. We estimated TDAC among these individuals by calculating a ferric reducing ability of plasma (FRAP) score based on data from food-frequency questionnaires. Body composition was assessed by means of dual X-ray absorptiometry at baseline and every subsequent 3–5 years. From these data, we calculated fat mass index (FMI), fat-free mass index (FFMI), android-to-gynoid fat ratio (AGR), body fat percentage (BF%) and body mass index (BMI). We also assessed hand grip strength at two time points and prevalence of sarcopenia at one time point in a subset of participants. Data were analyzed using linear mixed models or multinomial logistic regression models with multivariable adjustment. We found that higher FRAP score was associated with higher FFMI (0.091 kg/m^2^ per standard deviation (SD) higher FRAP score, 95% CI 0.031; 0.150), lower AGR (−0.028, 95% CI −0.053; −0.003), higher BMI (0.115, 95% CI 0.020; 0.209) and lower BF% (−0.223, 95% CI −0.383; −0.064) across follow-up after multivariable adjustment. FRAP score was not associated with hand grip strength or sarcopenia. Additional adjustment for adherence to dietary guidelines and exclusion of individuals with comorbid disease at baseline did not change our results. In conclusion, dietary intake of antioxidants may positively affect the amount of lean mass and overall body composition among the middle-aged and elderly.

## 1. Introduction

Dietary intake of antioxidants, a group of compounds that are capable of mitigating oxidative stress, has been shown to lower the risk of diseases such as type 2 diabetes, myocardial infarction and cancer [1,2,3]. Examples of such dietary antioxidants include vitamins C and E, polyphenols and carotenoids, and foods that are generally regarded as rich in antioxidants include fruits, vegetables, tea, coffee, spices and herbs [4,5]. Because multiple antioxidants may have synergistic effects, it is important to study the total dietary antioxidant capacity (TDAC) comprehensively rather than considering the effects of individual compounds [6]. Although the exact intermediate pathways through which the beneficial health effects of TDAC occur are not precisely known, there is some evidence that higher TDAC is inversely associated with the presence of obesity and age-related muscle loss [7,8,9].

In recent years, advances in imaging technology have allowed for more thorough assessment of body composition in population studies than was previously possible. In particular, the use of dual X-ray absorptiometry (DXA) allows for accurate estimation of body composition at low cost and negligible radiation exposure [10,11]. DXA not only provides information about total body fat mass and fat-free mass, but also about fat distribution (i.e., android or gynoid type fat distribution) within a given individual. Investigating these detailed measures of body composition as opposed to more simple measures such as body mass index (BMI) is of importance because fat mass and fat-free mass differentially affect risk of several different health outcomes [12]. Changes in body composition are especially relevant in elderly individuals, in whom loss of muscle mass and function is commonly observed [13]. Such losses in muscle mass are associated with reduced functional outcomes over time [14].

Most studies on antioxidants and body composition so far have been of cross-sectional design with relatively small sample sizes and have only investigated a small number of antioxidants. A systematic review reported that although a number of cross-sectional studies found a significant inverse association between TDAC and waist circumference, issues relating to the design or statistical power of these studies made inference on this association difficult [15]. No studies thus far have investigated a comprehensive measure of TDAC in relation to more detailed body composition measurements. For these reasons, we aimed to investigate the relationship between TDAC and longitudinal profiles of body composition derived by means of DXA, as well as muscle strength and sarcopenia, in the context of a large population-based cohort study among middle-aged and elderly individuals.

## 2. Materials and Methods

### 2.1. The Rotterdam Study

The general design of the Rotterdam Study has been outlined extensively elsewhere [16]. In short, this prospective cohort study was initiated in 1990 in the district of Ommoord, Rotterdam, the Netherlands. All inhabitants of this district aged 55 years or older (*n* = 10,215) were invited to participate, and 7983 participants were included for a response rate of 78% (subcohort RS-I). In 2000, a second subcohort of participants who had moved into the study district or had become 55 years of age since the start of the Rotterdam Study was included in the study; 4472 were invited and 3011 participated (response rate 67%) (subcohort RS-II). A third subcohort was added in 2006 with the inclusion of 3932 participants, out of 6057 invited (response rate 65%), aged 45–54 years (subcohort RS-III). Together, these subcohorts account for a total number of participants of 14,926 at baseline. Participants underwent home interviews and an extensive set of physical examinations at baseline and every subsequent 3–4 years. The Medical Ethics Committee of Erasmus University Medical Center (registration number MEC 02.1015) and the review board of the Dutch Ministry of Health, Welfare and Sports (Population Screening Act WBO, license number 1071272-159521-PG) have approved the Rotterdam Study. The Rotterdam Study has been entered into the Netherlands National Trial Register (NTR) and into the WHO International Clinical Trials Registry Platform (ICTRP) under shared catalogue number NTR6831. All participants have provided written informed consent to participate in the study and to have their information obtained from treating physicians [17].

### 2.2. Assessment Total Dietary Antioxidant Capacity

Assessment of dietary intake was performed at the fifth examination round for the first subcohort (RS-I-5; 2009–2011), the first examination round of the second subcohort (RS-II-1; 2000–2001), the third examination round of the second cohort (RS-II-3; 2011–2012) and the first examination round of the third subcohort (RS-III-1; 2006–2008) (Supplementary Figure S1). Semi-quantitative food frequency questionnaires (FFQs) were used to assess dietary intake at baseline. We used two versions: a 170-item FFQ for the measurements of RS-II-1 in 2000 and an updated 389-item FFQ for the later examination rounds, as described in detail elsewhere [18]. Food intake data from all cohorts was converted into daily nutrient and energy intake (in kcal) using Dutch Food Composition Tables corresponding to the years of dietary assessment. Both FFQs were developed to assess diet in a Dutch population and both FFQs have been validated against other assessment methods, which showed that the FFQs are able to adequately rank participants according to nutrient intakes. The 170-item FFQ was validated against fifteen twenty-four-hour food records and four twenty-four-hour urea excretion samples among 80 participants of the Rotterdam Study. Pearson’s correlations between the FFQ and the food records ranged between 0.44 and 0.85, and Spearman’s correlation for estimated protein intake with urea excretion samples was 0.67 [19]. The 389-item FFQ was validated among two other Dutch populations using a 9-day dietary record and a 4-week dietary history, with Pearson’s correlations ranging between 0.40 and 0.86 [20,21].

The TDAC was calculated for each participant using the Antioxidant Food Table published by Carlsen et al., who used a ferric reducing ability of plasma (FRAP) assay to estimate the antioxidant content of over 3100 types of food [22]. This assay measures absorption changes that occur when ferric ion (Fe^3+^) is reduced to ferrous ion (Fe^2+^) in the presence of antioxidants from different food samples. The measured value is the antioxidant capacity for a given type of food expressed in mmol per 100 g. We multiplied these values by the consumption of the different types of food in our FFQs and then summed across all food types for every participant. The resulting value is a FRAP score that represents the total dietary antioxidant intake in mmol per day. Because the Antioxidant Food Table lists different antioxidant capacities for the same types of food produced by different manufacturers, we consulted nutrition scientists from Wageningen University, the Netherlands, to determine the closest Dutch food equivalent for food types with multiple listings. Due to lack of data, food supplements were not included in the calculation of TDAC.

### 2.3. Measurement of Body Composition

Body composition was measured by means of Dual X-ray Absorptiometry (DXA; Prodigy and iDXA devices, GE Healthcare, Chicago, United States). From these DXA data, we calculated fat mass index (FMI) as total fat mass in kilograms divided by height in meters squared, fat-free mass index (FFMI) as total lean mass (excluding bone mineral content) in kilograms divided by height in meters squared, android-to-gynoid fat ratio (AGR) as android fat mass in kilograms divided by gynoid fat mass in kg and total body fat percentage (BF%) by expressing total fat mass in kilograms as a percentage of total body weight in kilograms. Weight was recorded with a digital scale with the participant wearing light clothing and height was recorded with the participant in a standing position without shoes. BMI was calculated as total body weight in kilograms divided by height in meters squared.

Sarcopenia was defined according to the updated European Working Group on Sarcopenia in Older People (EWGSOP2) criteria [23,24]. According to these criteria, sarcopenia is defined as the combination of low muscle strength and low muscle quantity or quality with or without low physical performance, and probable sarcopenia is defined as isolated low muscle strength. We defined low muscle strength as a peak hand grip strength <27 kg (for men) or <16 kg (for women) over three attempts as measured at the Rotterdam Study research center. Low muscle quantity was defined as appendicular skeletal muscle mass index (ASMI; appendicular skeletal muscle mass divided by height squared) <7.0 kg/m^2^ (for men) or <5.5 kg/m^2^ (for women). Appendicular skeletal muscle mass was assessed by DXA and was calculated as the sum of the muscle masses of all four limbs. Low physical performance was defined as a gait speed ≤0.8 m/s.

### 2.4. Population for Analysis

Data availability in the different examination rounds is outlined in Supplementary Figure S1. Full-body DXA measurements were performed from 2009 (RS-I-5), 2004 (RS-II-2) and 2006 (RS-III-1) onward, which constitute the baseline of our current study for a total of 8547 participants. Dietary data were available for 5791 of these 8457 individuals, of which 309 were excluded for having invalid dietary data (reported energy intake <500 or >5000 kcal/day). Of the remaining 5663 participants, 4971 underwent DXA at least once. Another 375 individuals were excluded because their body mass index (BMI) was greater than 35 kg/m^2^. Such individuals typically exceed the surface area limitations of the DXA-scanner, which would compromise image accuracy and therefore produce biased estimations of body composition [25]. Thus, our final population for analysis consisted of 4595 individuals, of whom 3065 had more than one DXA measurement available.

Data on hand grip strength were available for 4193 individuals from the total of 4595, measured from 2009 (RS-I-5), 2011 (RS-II-3) and 2006 (RS-III-1) onward. Sufficient data to assess the prevalence of sarcopenia was only available for the fifth visit round of the first cohort (RS-I-5) and the third visit round of the second cohort (RS-II-3) for a total of 2001 participants. For the second cohort (RS-II), we used dietary data from the first examination round (RS-II-1) for the DXA outcomes and dietary data from the third examination round (RS-II-3) for the analyses pertaining to hand grip strength and prevalent sarcopenia, to minimize the time between assessment of FRAP score and the respective outcomes (Supplementary Figure S1).

### 2.5. Covariates

The following variables were considered as potential confounders in our analyses: age, sex, Rotterdam Study cohort, hypertension status, presence of dyslipidemia, daily alcohol consumption, daily physical activity, smoking status, highest attained level of education, total daily energy intake, overall diet quality and serum glucose level. Potential confounders were selected based on general knowledge of their association with exposure and outcomes or on the basis of previous literature. We used directed acyclic graph (DAG) modeling to help theorize which variables would potentially be relevant to include in our analyses as confounders [26]. Participant height and weight were recorded at every center visit. Participants were considered to have dyslipidemia if their total serum cholesterol was >6.5 mmol/L or if they used lipid-lowering medication. Serum cholesterol was determined in blood samples taken at baseline using a CHOD-PAP method (Monotest Cholesterol kit, Boehringer Mannheim Diagnostica, Mannheim, Germany) [27]. Hypertension was defined as having a systolic blood pressure ≥140 mmHg, a diastolic blood pressure ≥90 mmHg or using antihypertensive medication. We performed two blood pressure readings at the right upper arm using a random-zero sphygmomanometer. Information on use of lipid-lowering or antihypertensive drugs was obtained during home interviews and by consulting pharmacy dispensing records. Smoking status (never, former or current user of tobacco products) and highest attained level of education were assessed during home interviews. Energy intake (kcal/day) and alcohol consumption (glasses/day) were derived from the FFQ data. To assess physical activity we used the LASA physical activity questionnaire and a modified version of the Zutphen Study Physical Activity Questionnaire to estimate activity in metabolic equivalent of task (MET) hours [28,29]. Because different questionnaires were used, we calculated cohort-specific standard deviation (SD) scores for physical activity. Finally, as a measure of overall healthiness of diet, we used a diet quality score which describes the degree of adherence to the Dutch Dietary Guidelines [18]. Data on comorbid disease (coronary heart disease, heart failure, stroke, type 2 diabetes and cancer) were collected by consulting general practitioners’ records and hospital discharge data and using measurements in our research center [30,31,32,33].

### 2.6. Statistical Analysis

In order to assess the association between baseline FRAP score and longitudinal changes in body composition measures and hand grip strength, we used a linear mixed model approach. We used the residual method to adjust FRAP score for energy intake [34]. We did this in each of the cohorts separately to account for the use of different FFQs and we used the standardized residuals as exposure in our analyses. For every regression model, we investigated whether non-linear terms (polynomials or three-knot natural cubic splines) for the variables age and time significantly (*p* < 0.05) improved model fit by performing likelihood ratio tests with the models fitted under maximum likelihood. Using the same procedure, we tested whether interaction between FRAP score and time, age or sex significantly improved the model fit. If this was the case, the non-linear or interaction terms were kept in the model. For the random effects structure of these models, we specified random intercepts and random slopes (for time between repeated measurements). In order to investigate the association between FRAP score and prevalence of probable sarcopenia or sarcopenia, we fitted multinomial logistic regression models. To provide insight into how the covariates influence the association between FRAP score and body composition parameters, these covariates were introduced into the models in a stepwise process. Model 1 was adjusted for age, sex, Rotterdam Study cohort and time difference between exposure and outcome measurement (where applicable) in years. Model 2 was additionally adjusted for hypertension, dyslipidemia, alcohol consumption (natural log-transformed), physical activity, smoking, education and serum glucose. In model 3, we also included diet quality. Missing values were accounted for by the use of ten-fold multiple imputation. All statistical analyses were performed using R version 3.6.1 (The R Foundation for Statistical Computing, Vienna, Austria), using the mice package (version 3.8.0) for multiple imputation and the nlme package (version 3.1-140) for designing the linear mixed models [35,36]. As sensitivity analysis, we repeated our analyses excluding participants with comorbidities (as defined previously) at baseline.

## 3. Results

The characteristics of the study population are presented in Table 1. Overall, the mean FRAP score was 25.2 (SD 10.3) mmol/day. For the different measures of body composition, population averages at the first measurement were 9.3 (2.9) kg/m^2^ for FMI, 17.5 (2.1) kg/m^2^ for FFMI, 0.6 (0.2) for AGR, 34.0 (7.9) for BF% and 26.8 (3.4) for BMI. The food groups that contributed most to FRAP in our study were coffee, fruit, vegetables and tea. For those participants with more than one DXA measurement (*n* = 3065), the average follow-up duration was 6.6 years (6.1 years for those with two measurements (*n* = 2705) and 10.9 years for those with 3 measurements (*n* = 360)).

The results of our main analyses are displayed in Table 2. We observed an inverse association between FRAP score and FMI during follow-up in model 1, but this association was explained by the metabolic and lifestyle factors in model 2 and by overall diet quality in model 3 (model 3: −0.018 kg/m^2^ per SD higher FRAP score, 95% CI −0.089; 0.053). Furthermore, we found a positive association between FRAP score and FFMI during follow-up in model 1, for which the effect estimate hardly changed and remained statistically significant after adjustment for covariates (model 3: 0.091, 95% CI 0.031; 0.150). We observed an inverse association of FRAP score with AGR, which was also persistent across models (model 3: −0.028, 95% CI −0.053; −0.003). FRAP score was not significantly associated with BMI during follow-up in the first model, but we did observe a positive association in model 3 (0.115, 95% CI 0.020; 0.209). Finally, we found that FRAP score was inversely associated with BF% during follow-up, with some attenuation after adjustment for covariates (model 3: −0.223, 95% CI −0.383; −0.064).

The results for subsequent analyses on hand grip strength and sarcopenia are presented in Table 3 and Table 4. FRAP score was not associated with hand grip strength across follow-up after multivariable adjustment (model 3: 0.177, 95% CI −0.135; 0.488) (Table 3). Among the subgroup of individuals with data on sarcopenia, we identified 314 cases of probable sarcopenia and 104 cases of sarcopenia. FRAP score was not associated with probable sarcopenia (model 3: OR 0.95, 95% CI 0.81; 1.12) (Table 4). Although higher FRAP score was associated with lower probability of sarcopenia in model 1 (OR 0.77; 95% CI 0.60; 0.99), after adjustment for covariates this association slightly attenuated and was no longer statistically significant (model 3: OR 0.81, 95% CI 0.62; 1.05).

Excluding participants with one or more comorbidities at baseline left 3327 individuals for analysis. Repeating our analyses in this subgroup did not substantially change our conclusions with regard to the association between TDAC and body composition, although we did observe some attenuation of the association between FRAP score and AGR (Supplemental Tables S1–S3). We observed no significant interaction between FRAP score and follow-up time for any of the body composition outcomes in our analyses, indicating that FRAP generally does not modify the rate at which body composition changes over time. We did observe significant interaction between FRAP score and time on hand grip strength (*p* for interaction 0.003), suggesting that FRAP modifies the rate at which hand grip strength changes over time. We found significant interaction between FRAP score and sex only on FFMI (*p* for interaction 0.043) and between FRAP score and age only on AGR (*p* for interaction 0.046). Considering these findings, we additionally stratified all our analyses by sex and median age at baseline (Supplemental Tables S4–S9). We observed that FRAP score was more strongly associated with FFMI in women (0.189, 95% CI 0.135; 0.243) compared to men (0.070, 95% CI 0.007; 0.133) after adjustment for all covariates, but these sex differences were generally not reflected in the other outcome parameters. Similarly, while FRAP score was more strongly associated with AGR in younger participants (−0.007, 95% CI −0.012; −0.001) compared to older participants (−0.0004, 95% CI −0.006; 0.006) after adjustment, this pattern was not reflected in the other outcome parameters.

## 4. Discussion

In this prospective cohort study, higher total dietary antioxidant capacity (TDAC) was associated with higher fat-free mass index (FFMI), higher body mass index (BMI), lower body fat percentage (BF%) and lower android-to-gynoid fat ratio (AGR) across follow-up. We found no association between TDAC and the presence of sarcopenia, probable sarcopenia or hand grip strength. The observed associations were independent of degree of adherence to dietary guidelines. Overall, this combination of findings from our study indicates a positive association between TDAC and fat-free mass in particular. TDAC was positively associated with FFMI but was not associated with FMI. Hence, the decrease in body fat percentage we observe with higher TDAC is likely mainly due to higher fat-free mass rather than lower fat mass.

Several previous studies have examined the association between individual compounds with antioxidative properties and indicators of body composition. For example, a cross-sectional study of 3182 participants found that serum levels of β-carotene and vitamin C, but not vitamin E, zinc or selenium, were lower in participants with higher BMI [37]. Another cross-sectional study on a similar scale found that serum levels of magnesium, a cofactor for a number of antioxidant enzymes, were associated with lower BMI and waist circumference [38]. Several studies have also been performed that examined TDAC in relation to anthropometric measures. A systematic review reported that TDAC was examined in relation to waist circumference in several studies, two of which found a significant (inverse) association [7,15,39]. One of these two studies investigated TDAC in relation to abdominal obesity (defined as a waist circumference ≥ 95 cm) measured 3 years after baseline among 1983 young adults, and reported lower occurrence of abdominal obesity across quartiles of TDAC after multivariable adjustment [7]. The other study reported lower waist circumference with higher trolox-equivalent antioxidant capacity (TEAC) among 266 young adults in a cross-sectional analysis adjusted only for energy intake and sex [39]. Another cross-sectional study found an association between measures of TDAC and obesity as measured by BMI, but not between TDAC and waist circumference [40]. Differences between studies with regards to the observed associations could be accounted for by differences in sample size, as a number of previous studies had considerably fewer participants available than ours and other larger studies [7,8,39]. Furthermore, a number of previous studies also did not adjust their analyses for cardiometabolic risk factors [39], or had a population that was demographically and ethnically different from ours [7,8,40]. Previous studies also differed with regards to the measure of TDAC that was investigated [39,40,41]. Notably, no studies thus far have investigated TDAC in relation to more detailed measures of obesity derived from DXA data. This is important considering that BMI alone fails to fully capture inter-individual differences in fat and lean mass [42]. Furthermore, when used as a measurement of adiposity, waist circumference may underestimate the association between adiposity and cardiometabolic risk factors when compared to DXA-derived measurements of adiposity [43]. These limitations emphasize the importance of studying more comprehensive measures of body composition over simple anthropometrics.

The positive association between TDAC and fat-free mass we observed in our study could be mediated by the reduction in oxidative stress levels that is associated with antioxidant consumption [44]. One of the major sources of oxidative stress is the presence of excess reactive oxygen species (ROS), which are chemically reactive molecules naturally produced in response to cellular stress and inflammatory processes [45]. While ROS have certain physiological functions at low concentrations, excess ROS production in response to stressors has adverse effects on cellular functioning [45]. High levels of ROS may specifically affect skeletal muscle mass and strength through a number of pathways [46]. For example, oxidative stress induces activation of proteolytic compounds and mediates the release of pro-inflammatory cytokines, which may lead to protein degradation and atrophy or loss of muscle fibers [47]. Previous studies have also demonstrated that aging is associated with higher levels of ROS in skeletal muscle [48,49]. These adverse effects of ROS on muscle tissue, potentially exacerbated by increasing levels of ROS with aging, may in part be responsible for the commonly observed loss of muscle mass in the elderly [47,50]. Given that antioxidants have the ability to lower oxidative stress levels, a high consumption of antioxidants might reduce the extent to which these deleterious processes take place [45]. Increased consumption of dietary antioxidants may also help counteract the age-related deficiencies in the endogenous antioxidant defense system that have been reported in the elderly [51]. In spite of our observation that higher TDAC was associated with higher FFMI, we did not observe an association between TDAC and hand grip strength. This indicates that the increased muscle mass that is associated with higher TDAC is not also paired with increased muscle strength (Table 3). This discrepancy between findings for muscle mass and muscle strength could be explained by the fact that despite the correlation between these parameters, muscle strength may also be determined by neural factors in addition to muscle mass alone [52]. Furthermore, in a previous study, it was demonstrated that muscle mass accounted for only 13% of the variation in muscle strength among older adults [53]. We observed no association between TDAC and probability of probable sarcopenia or sarcopenia after adjustment for covariates. Sarcopenia is a complex and heterogeneous condition that can be defined according to different combinations of criteria within the EWGSOP2 definition [23]. Possibly, other factors than TDAC play a more prominent role in the pathogenesis of sarcopenia. We also had limited statistical power in this analysis due to the relatively low number of sarcopenia cases (*n* = 104) available.

In addition, although we found that the association between TDAC and FFMI appeared to be somewhat stronger in women compared to men, previous literature has not provided consistent evidence of sex differences with relation to this association or the associations between individual antioxidants and anthropometrics [37,54]. However, in the case of our study, these sex differences could also be explained by differences in statistical power between the groups considering that we had more women (*n* = 2581) than men (*n* = 2014) available for analysis. The association between TDAC and android-to-gynoid fat ratio, and the variation of the strength of this association with age, has not been previously reported in the literature. Further research is needed in order to elucidate these findings.

The strengths of our study include its prospective design with repeated assessment of body composition over a period of, on average, more than six years. In addition, we had a large population available for analysis. We investigated a comprehensive measure of TDAC, which takes into account the potential synergistic effects of all antioxidants that are contained in the diet, rather than focusing on single antioxidative compounds. In addition, we analyzed advanced measures of body composition in our study as opposed to only anthropometrics, enabling us to study the association between TDAC and body composition in greater detail than was previously possible. Furthermore, we were also able to adjust for a large number of covariates related to lifestyle, cardiometabolic status and dietary habits. Although it is possible that high TDAC could reflect an overall healthy diet because healthy foods are generally rich in antioxidants, we were able to demonstrate that our results persisted after adjustment for adherence to guidelines for a healthy diet. Several limitations should be taken into account when interpreting our findings. First, we estimated TDAC based on a Norwegian database listing the antioxidant content of different types of food [22]. It is possible that differences with regards to country of origin, growth conditions and processing of food have led to some error in the estimation of TDAC, although we did attempt to mitigate this by determining the closest Dutch food equivalent for products with multiple listings in the database. Second, we had no information available on the cooking methods used by participants. It has been demonstrated that cooking methods may also affect the antioxidant content of food [55]. Third, we had no data available on the use of food supplements in our study, so these could not be taken into account in our estimation of the TDAC. Fourth, the FFQ we used in order to assess dietary habits may inherently provide some measurement error, although our FFQ were both validated and shown to be adequate in ranking according to nutrient intake [19,20]. Fifth, we had a relatively limited number of repeated measurements available per participant, which may in turn limit the accuracy of the estimated longitudinal body composition profiles.

In conclusion, higher total dietary antioxidant capacity was associated with higher fat-free mass index in this longitudinal population-based cohort study of over 4500 middle-aged and elderly participants. Our findings indicate that increased consumption of antioxidants may have favorable effects on body composition and may play a role in preserving lean mass over time.

## Figures and Tables

**Table 1:** Baseline characteristics of the total study population (*n* = 4595).

Age (Years)	65.1 (10.8)
Sex	
Female	2581 (56.2%)
Male	2014 (43.8%)
Highest level of education (%)	
Primary	372 (8.1%)
Lower/intermediate general or lower vocational	1782 (38.8%)
Intermediate vocational or higher general	1063 (23.1%)
Higher vocational or university	1063 (23.1%)
Hypertension (%)	
No	1668 (36.3%)
Yes	2927 (63.7%)
Dyslipidemia (%)	
No	2644 (57.5%)
Yes	1951 (42.5%)
Alcohol intake (glasses/day) ^a^	0.9 [1.1]
Smoking (%)	
Never smoker	1429 (31.1%)
Former smoker	2293 (49.9%)
Current smoker	873 (19.0%)
Physical activity (MET-hours/week) ^a^	54.3 [67.7]
Energy intake (kcal/day)	2199 (676)
Dietary guideline score	6.8 (1.9)
Fasting serum glucose (mmol/L)	5.6 (1.2)
Height (cm) ^b^	168.6 (9.3)
Weight (kg) ^b^	76.4 (12.6)
Body mass index (kg/m^2^) ^b^	26.8 (3.4)
Fat mass index (kg/m^2^) ^b^	9.3 (2.9)
Fat-free mass index (kg/m^2^) ^b^	17.5 (2.1)
Android-to-gynoid fat ratio ^b^	0.6 (0.2)
Total body fat percentage (%) ^b^	34.0 (7.9)
FRAP score (mmol/day)	25.2 (10.3)

Variables are presented as mean (SD) unless otherwise indicated. ^a^ Median (interquartile range). The presented statistics represent the data after ten-fold multiple imputation. ^b^ Variable is presented for the individuals who participated in the baseline DXA measurement round (*n* = 3770), i.e., RS-I-5, RS-II-2 or RS-III-1.

**Table 2:** Longitudinal associations between Ferric Reducing Ability of Plasma (FRAP) score and fat mass index, fat-free mass index, android-to-gynoid fat ratio, body mass index and body fat percentage.

	Fat Mass Index (kg/m^2^)	*p*-Value	Fat-Free Mass Index (kg/m^2^)	*p*-Value	Android-To-Gynoid Fat Ratio	*p*-Value	Body Mass Index (kg/m^2^)	*p*-Value	Body Fat %	*p*-Value
Model 1 ^a^	−0.083 (−0.156; −0.010)	0.026	0.076 (0.016; 0.135)	0.013	−0.028 (−0.054; −0.002)	0.033	0.039 (−0.058; 0.137)	0.426	−0.365 (−0.527; −0.202)	<0.001
Model 2 ^b^	−0.037 (−0.108; 0.033)	0.301	0.088 (0.028; 0.147)	0.004	−0.029 (−0.054; −0.004)	0.025	0.092 (−0.002; 0.186)	0.055	−0.266 (−0.425; −0.107)	0.001
Model 3 ^c^	−0.018 (−0.089; 0.053)	0.619	0.091 (0.031; 0.150)	0.003	−0.028 (−0.053; −0.003)	0.026	0.115 (0.020; 0.209)	0.017	−0.223 (−0.383; −0.064)	0.006

N = 4595. Results are presented as regression coefficient (β) with corresponding 95% CI per 1 standard deviation increment in FRAP. ^a^ Model 1: adjusted for time interval, age, sex and Rotterdam Study cohort. ^b^ Model 2: additionally adjusted for hypertension status, presence of dyslipidemia, daily alcohol consumption, daily physical activity, smoking status, highest attained level of education and serum glucose. ^c^ Model 3: additionally adjusted for adherence to dietary guideline score. At the baseline examination round (cohorts RS-I-5, II-2 and III-1), 3770 participants underwent a DXA measurement; during the second round (cohorts I-6, II-3 and III-2), 3492 participants were measured and, during the final examination round (cohort II-4), 718 participants were measured. Of the 4595 total participants, 1530 participants were measured once during the study period, 2705 were measured twice and 360 were measured at all three time points.

**Table 3:** Longitudinal associations between Ferric Reducing Ability of Plasma (FRAP) score and hand grip strength.

	Hand Grip Strength (kg) (*n* = 4193)	*p*-Value
Model 1 ^a^	0.232 (−0.078; 0.541)	0.142
Model 2 ^b^	0.182 (−0.129; 0.493)	0.251
Model 3 ^c^	0.177 (−0.135; 0.488)	0.267

Results are presented as regression coefficient (β) with corresponding 95% CI per 1 standard deviation increment in FRAP. ^a^ Model 1: adjusted for time interval, age and sex. ^b^ Model 2: additionally adjusted for hypertension status, presence of dyslipidemia, daily alcohol consumption, daily physical activity, smoking status, highest attained level of education and serum glucose. ^c^ Model 3: additionally adjusted for adherence to dietary guideline score.

**Table 4:** Associations between Ferric Reducing Ability of Plasma (FRAP) score and prevalence of (probable) sarcopenia.

	Probable Sarcopenia (*n* Cases = 314)	*p*-Value	Sarcopenia (*n* Cases = 104)	*p*-Value
Model 1 ^a^	0.93 (0.79; 1.08)	0.342	0.77 (0.60; 0.99)	0.045
Model 2 ^b^	0.95 (0.81; 1.11)	0.504	0.80 (0.62; 1.04)	0.098
Model 3 ^c^	0.95 (0.81; 1.12)	0.564	0.81 (0.62; 1.05)	0.110

N = 2001. Results are presented as odds ratio (OR) with corresponding 95% CI per 1 standard deviation increment in FRAP. ^a^ Model 1: adjusted for age, sex and Rotterdam Study cohort. ^b^ Model 2: additionally adjusted for hypertension status, presence of dyslipidemia, daily alcohol consumption, daily physical activity, smoking status, highest attained level of education and serum glucose. ^c^ Model 3: additionally adjusted for adherence to dietary guideline score.

## Supplementary Materials

The following are available online at https://www.mdpi.com/2076-3921/9/8/728/s1, Figure S1: Overview of Rotterdam Study Measurement Rounds, Table S1: Longitudinal associations between Ferric Reducing Ability of Plasma (FRAP) score and fat mass index, fat-free mass index, android-to-gynoid fat ratio, body mass index and body fat percentage; excluding participants with selected comorbidities at baseline. Table S2: Longitudinal associations between Ferric Reducing Ability of Plasma (FRAP) score and hand grip strength; excluding participants with selected comorbidities at baseline. Table S3: Associations between Ferric Reducing Ability of Plasma (FRAP) score and (probable) sarcopenia; excluding participants with selected comorbidities at baseline. Table S4: Longitudinal associations between Ferric Reducing Ability of Plasma (FRAP) score and fat mass index, fat-free mass index, android-to-gynoid fat ratio, body mass index and body fat percentage; stratified by sex. Table S5: Longitudinal associations between Ferric Reducing Ability of Plasma (FRAP) score and hand grip strength; stratified by sex. Table S6: Longitudinal associations between Ferric Reducing Ability of Plasma (FRAP) score and (probable) sarcopenia; stratified by sex. Table S7: Longitudinal associations between Ferric Reducing Ability of Plasma (FRAP) score and fat mass index, fat-free mass index, android-to-gynoid fat ratio, body mass index and body fat percentage; stratified by age. Table S8: Associations between Ferric Reducing Ability of Plasma (FRAP) score and hand grip strength, stratified by age. Table S9: Associations between Ferric Reducing Ability of Plasma (FRAP) score and (probable) sarcopenia, stratified by age.
