# Supplementary material for: Total Dietary Antioxidant Capacity and Longitudinal Trajectories of Body Composition
Source: Antioxidants (Basel). 2020 Aug 10;9(8):728. doi: 10.3390/antiox9080728 (PMC7465193; doi:10.3390/antiox9080728)
Supplement: Supplementary file 1 [file antioxidants-09-00728-s001.zip › supp_tables_vds.docx]

**Supp. Table 1. Longitudinal associations between Ferric Reducing Ability of Plasma (FRAP) score and fat mass index, fat-free mas index, android-to-gynoid fat ratio, body mass index and body fat percentage; excluding participants with selected comorbidities at baseline.**

|  | **Fat mass index (kg/m2)** | p-value | **Fat free mass index (kg/m2)** | p-value | **Android-to-gynoid fat ratio** | p-value | **Body mass index (kg/m2)** | p-value | **Body fat %** | p-value |
| --- | --- | --- | --- | --- | --- | --- | --- | --- | --- | --- |
| Model 1^a^ | -0.100  (-0.185 ; -0.015) | **0.022** | 0.107  (0.038 ; 0.177) | **0.003** | -0.021  (-0.052 ; 0.010) | 0.182 | 0.058  (-0.055 ; 0.170) | 0.314 | 0.458  (-0.649 ; -0.266) | **< 0.001** |
| Model 2^b^ | -0.049  (-0.131 ; 0.033) | 0.243 | 0.123  (0.054 ; 0.192) | **0.001** | -0.026  (-0.056 ; 0.004) | 0.093 | 0.117  (0.009 ; 0.226) | **0.035** | -0.350  (-0.536 ; -0.163) | **< 0.001** |
| Model 3^c^ | -0.028  (-0.111 ; 0.054) | 0.500 | 0.126  (0.056 ; 0.195) | **< 0.001** | -0.026  (-0.056 ; 0.004) | 0.087 | 0.141  (0.032 ; 0.250) | **0.011** | -0.303  (-0.490 ; -0.116) | **0.001** |

Total number of participants with one or more prevalent comorbidities was 1268 (366 cases of cancer other than non-melanoma skin cancer, 531 cases of type 2 diabetes, 113 cases of heart failure, 309 cases of coronary heart disease and 153 cases of a history of stroke), leaving 3327 participants free of these comorbidities at baseline. Results are presented as regression coefficient (β) with corresponding 95% CI per 1 standard deviation increment in FRAP score. ^a^Model 1: adjusted for time interval, age, sex and Rotterdam Study cohort. ^b^Model 2: additionally adjusted for hypertension status, presence of dyslipidemia, daily alcohol consumption, daily physical activity, smoking status, highest attained level of education and serum glucose. ^c^Model 3: additionally adjusted for adherence to dietary guideline score.

|  |  | **Hand grip strength (kg)**  **(n = 3070)** | p-value |
| --- | --- | --- | --- |
| Model 1^a^ |  | 0.207  (-0.161 ; 0.574) | 0.270 |
| Model 2^b^ |  | 0.180  (-0.190 ; 0.550) | 0.341 |
| Model 3^c^ |  | 0.182  (-0.188 ; 0.553) | 0.335 |

**Supplemental Table 2. Longitudinal associations between Ferric Reducing Ability of Plasma (FRAP) score and hand grip strength; excluding participants with selected comorbidities at baseline.**

Total number of participants with one or more prevalent comorbidities was 1123 (323 cases of cancer other than non-melanoma skin cancer, 458 cases of type 2 diabetes, 102 cases of heart failure, 276 cases of coronary heart disease and 132 cases of a history of stroke), leaving 3070 participants free of these comorbidities at baseline. Results are presented as regression coefficient (β) with corresponding 95% CI per 1 standard deviation increment in FRAP score. ^a^Model 1: adjusted for time interval, age, sex and Rotterdam Study cohort. ^b^Model 2: additionally adjusted for hypertension status, presence of dyslipidemia, daily alcohol consumption, daily physical activity, smoking status, highest attained level of education and serum glucose. ^c^Model 3: additionally adjusted for adherence to dietary guideline score.

|  | **Probable sarcopenia**  **(n cases = 166)** | p-value | **Sarcopenia**  **(n cases = 51)** | p-value |
| --- | --- | --- | --- | --- |
| Model 1^a^ | 0.93  (0.75 ; 1.14) | 0.477 | 0.95  (0.67 ; 1.35) | 0.767 |
| Model 2^b^ | 0.95  (0.76 ; 1.18) | 0.627 | 0.94  (0.65 ; 1.35) | 0.736 |
| Model 3^c^ | 0.96  (0.77 ; 1.20) | 0.720 | 0.92  (0.64 ; 1.34) | 0.673 |

**Supplemental Table 3. Associations between Ferric Reducing Ability of Plasma (FRAP) score and (probable) sarcopenia; excluding participants with selected comorbidities at baseline.**

Total number of participants with one or more prevalent comorbidities was 782 (227 cases of cancer other than non-melanoma skin cancer, 301 cases of type 2 diabetes, 89 cases of heart failure, 198 cases of coronary heart disease and 95 cases of a history of stroke), leaving 1219 participants free of these comorbidities at baseline. Results are presented as regression coefficient (β) with corresponding 95% CI per 1 standard deviation increment in FRAP score. ^a^Model 1: adjusted for time interval, age, sex and Rotterdam Study cohort. ^b^Model 2: additionally adjusted for hypertension status, presence of dyslipidemia, daily alcohol consumption, daily physical activity, smoking status, highest attained level of education and serum glucose. ^c^Model 3: additionally adjusted for adherence to dietary guideline score.

(continued below)

**Supplemental Table 4. Longitudinal associations between Ferric Reducing Ability of Plasma (FRAP) score and fat mass index, fat-free mas index, android-to-gynoid fat ratio, body mass index and body fat percentage; stratified by sex.**

|  |  | **Model 1^a^** | **p-value** | **Model 2^b^** | **p-value** | **Model 3^c^** | **p-value** |
| --- | --- | --- | --- | --- | --- | --- | --- |
| Fat mass index  (kg/m^2^)  *P for interaction 0.348* | Men | -0.116  (-0.209 ; -0.024) | **0.014** | -0.095  (-0.185 ; -0.006) | **0.037** | -0.079  (-0.169 ; 0.010) | 0.082 |
|  | Women | -0.054  (-0.163 ; 0.056) | 0.338 | 0.011  (-0.095 ; 0.118) | 0.835 | 0.037  (-0.070 ; 0.144) | 0.502 |
| Fat-free mas index  (kg/m^2^)  *P for interaction 0.043* | Men | 0.063  (0.001 ; 0.125) | **0.047** | 0.070  (0.007 ; 0.132) | **0.029** | 0.070  (0.007 ; 0.133) | **0.029** |
|  | Women | 0.180  (0.126 ; 0.235) | **< 0.001** | 0.183  (0.129 ; 0.237) | **< 0.001** | 0.189  (0.135 ; 0.243) | **< 0.001** |
| Android-to-gynoid fat ratio  *P for interaction 0.701* | Men | -0.030  (-0.071 ; 0.012) | 0.161 | -0.029  (-0.069 ; 0.012) | 0.166 | -0.028  (-0.068 ; 0.013) | 0.181 |
|  | Women | -0.026  (-0.059 ; 0.006) | 0.109 | -0.026  (-0.057 ; 0.005) | 0.103 | -0.026  (-0.057 ; 0.005) | 0.100 |
| Body Mass Index (kg/m^2^)  *P for interaction 0.117* | Men | -0.058  (-0.185 ; 0.069) | 0.370 | -0.028  (-0.152 ; 0.095) | 0.653 | -0.012  (-0.136 ; 0.112) | 0.850 |
|  | Women | 0.126  (-0.018 ; 0.269) | 0.087 | 0.194  (0.055 ; 0.332) | **0.006** | 0.223  (0.083 ; 0.363) | **0.002** |
| Body Fat %  *P for interaction 0.914* | Men | -0.354  (-0.572 ; -0.119) | **0.003** | -0.301  (-0.522 ; -0.080) | **0.008** | -0.261  (-0.482 ; -0.040) | **0.021** |
|  | Women | -0.377  (-0.607 ; -0.146) | **0.001** | -0.234  (-0.460 ; -0.008) | **0.043** | -0.181  (-0.409 ; 0.046) | 0.119 |

N = 2014 men and N = 2581 women. Results are presented as regression coefficient (β) with corresponding 95% CI per 1 standard deviation increment in FRAP score. ^a^Model 1: adjusted for time interval, age and Rotterdam Study cohort. ^b^Model 2: additionally adjusted for hypertension status, presence of dyslipidemia, daily alcohol consumption, daily physical activity, smoking status, highest attained level of education and serum glucose. ^c^Model 3: additionally adjusted for adherence to dietary guideline score. Among women, we observed no differences in the strength of the association between FRAP score and FFMI according to menopausal status (among a subset of women with data on menopausal status, n = 2077 of whom n = 1778 post-menopausal; p for interaction 0.365) or age (p for interaction 0.894).

**Supplemental Table 5. Longitudinal associations between Ferric Reducing Ability of Plasma (FRAP) score and hand grip strength; stratified by sex.**

|  |  | **Model 1^a^** | **p-value** | **Model 2^b^** | **p-value** | **Model 3^c^** | **p-value** |
| --- | --- | --- | --- | --- | --- | --- | --- |
| Hand grip strength  (kg)  *P for interaction 0.082* | Men | -0.229  (-0.654 ; 0.195) | 0.290 | -0.286  (-0.716 ; 0.144) | 0.192 | -0.289  (-0.723 ; -0.145) | 0.192 |
|  | Women | 0.184  (-0.076 ; 0.445) | 0.166 | 0.090  (-0.174 ; 0.353) | 0.504 | 0.062  (-0.205 ; 0.329) | 0.649 |

N = 1835 men and N = 2358 women. Results are presented as regression coefficient (β) with corresponding 95% CI per 1 standard deviation increment in FRAP. ^a^Model 1: adjusted for time interval, age and Rotterdam Study cohort. ^b^Model 2: additionally adjusted for hypertension status, presence of dyslipidemia, daily alcohol consumption, daily physical activity, smoking status, highest attained level of education and serum glucose. ^c^Model 3: additionally adjusted for adherence to dietary guideline score.

**Supplemental Table 6. Longitudinal associations between Ferric Reducing Ability of Plasma (FRAP) score and (probable) sarcopenia; stratified by sex.**

|  |  | **Model 1^a^** | **p-value** | **Model 2^b^** | **p-value** | **Model 3^c^** | **p-value** |
| --- | --- | --- | --- | --- | --- | --- | --- |
| Probable sarcopenia  (n cases = 314)  *P for interaction 0.546* | Men  (n cases = 137) | 0.98  (0.78 ; 1.24) | 0.884 | 0.98  (0.77 ; 1.24) | 0.872 | 0.99  (0.78 ; 1.25) | 0.924 |
|  | Women  (n cases = 177) | 0.88  (0.71 ; 1.09) | 0.233 | 0.93  (0.74 ; 1.15) | 0.492 | 0.93  (0.74 ; 1.16) | 0.506 |
| Sarcopenia  (n cases = 104)  *P for interaction 0.931* | Men  (n cases = 37) | 0.87  (0.57 ; 1.33) | 0.534 | 0.90  (0.58 ; 1.39) | 0.630 | 0.90  (0.58 ; 1.39) | 0.637 |
|  | Women  (n cases = 67) | 0.71  (0.52 ; 0.98) | **0.040** | 0.74  (0.53 ; 1.03) | 0.076 | 0.75  (0.54 ; 1.06) | 0.101 |

N = 909 men and N = 1092 women. Results are presented as odds ratios (OR) with corresponding 95% CI per 1 standard deviation increment in FRAP. ^a^Model 1: adjusted for age and Rotterdam Study cohort. ^b^Model 2: additionally adjusted for hypertension status, presence of dyslipidemia, daily alcohol consumption, daily physical activity, smoking status, highest attained level of education and serum glucose. ^c^Model 3: additionally adjusted for adherence to dietary guideline score.

**Supplemental Table 7. Longitudinal associations between Ferric Reducing Ability of Plasma (FRAP) score and fat mass index, fat-free mas index, android-to-gynoid fat ratio, body mass index and body fat percentage; stratified by age.**

|  |  | **Model 1^a^** | **p-value** | **Model 2^b^** | **p-value** | **Model 3^c^** | **p-value** |
| --- | --- | --- | --- | --- | --- | --- | --- |
| Fat mass index  (kg/m^2^)  *P for interaction 0.889* | Age <= 62.3 years | -0.095  (-0.199 ; 0.009) | 0.072 | -0.029  (-0.131 ; 0.072) | 0.570 | -0.009  (-0.110 ; 0.093) | 0.867 |
|  | Age > 62.3 years | -0.068  (-0.170 ; 0.034) | 0.192 | -0.036  (-0.135 ; 0.063) | 0.475 | -0.018  (-0.118 ; 0.081) | 0.720 |
| Fat-free mas index  (kg/m^2^)  *P for interaction 0.691* | Age <= 62.3 years | 0.107  (0.051 ; 0.163) | **< 0.001** | 0.116  (0.059 ; 0.172) | **< 0.001** | 0.117  (0.060 ; 0.174) | **< 0.001** |
|  | Age > 62.3 years | 0.133  (0.072 ; 0.193) | **< 0.001** | 0.135  (0.075 ; 0.194) | **< 0.001** | 0.142  (0.081 ; 0.202) | **< 0.001** |
| Android-to-gynoid fat ratio  *P for interaction 0.046* | Age <= 62.3 years | -0.008  (-0.014 ; -0.002) | **0.009** | -0.008  (-0.013 ; -0.002) | **0.009** | -0.007  (-0.012 ; -0.001) | **0.023** |
|  | Age > 62.3 years | -0.002  (-0.008 ; 0.004) | 0.524 | -0.001  (-0.007 ; 0.004) | 0.625 | -0.0004  (-0.006 ; 0.006) | 0.884 |
| Body Mass Index (kg/m^2^)  *P for interaction 0.794* | Age <= 62.3 years | 0.010  (-0.126 ; 0.146) | 0.880 | 0.087  (-0.045 ; 0.220) | 0.196 | 0.107  (-0.025 ; 0.240) | 0.113 |
|  | Age > 62.3 years | 0.065  (-0.074 ; 0.204) | 0.360 | 0.098  (-0.036 ; 0.233) | 0.150 | 0.122  (-0.013 ; 0.258) | 0.076 |
| Body Fat %  *P for interaction 0.942* | Age <= 62.3 years | -0.356  (-0.588 ; -0.124) | **0.003** | -0.220  (-0.449 ; 0.008) | 0.059 | -0.169  (-0.398 ; 0.060) | 0.148 |
|  | Age > 62.3 years | -0.361  (-0.587 ; -0.135) | **0.002** | -0.285  (-0.505 ; -0.066) | **0.011** | -0.251  (-0.472 ; -0.029) | **0.027** |

N = 2298 participants aged <= 62.3 (sample median) years and N = 2297 participants aged > 62.3 years. Results are presented as regression coefficient (β) with corresponding 95% CI per 1 standard deviation increment in FRAP. ^a^Model 1: adjusted for time interval, age, sex and Rotterdam Study cohort. ^b^Model 2: additionally adjusted for hypertension status, presence of dyslipidemia, daily alcohol consumption, daily physical activity, smoking status, highest attained level of education and serum glucose. ^c^Model 3: additionally adjusted for adherence to dietary guideline score.

**Supplemental Table 8. Associations between Ferric Reducing Ability of Plasma (FRAP) score and hand grip strength, stratified by age.**

|  |  | **Model 1^a^** | **p-value** | **Model 2^b^** | **p-value** | **Model 3^c^** | **p-value** |
| --- | --- | --- | --- | --- | --- | --- | --- |
| Hand grip strength  (kg)  *P for interaction 0.546* | Age <= 65.9 years | -0.167  (-0.601 ; 0.266) | 0.449 | -0.252  (-0.690 ; 0.186) | 0.260 | -0.247  (-0.687 ; 0.193) | 0.271 |
|  | Age > 65.9 years | 0.675  (0.234 ; 1.116) | **0.003** | 0.626  (0.185 ; 1.068) | **0.005** | 0.584  (0.140 ; 1.029) | **0.010** |

N = 2097 participants aged <= 65.9 (sample median) years and N = 2096 participants aged > 65.9 years. Results are presented as regression coefficient (β) with corresponding 95% CI per 1 standard deviation increment in FRAP. ^a^Model 1: adjusted for time interval, age, sex and Rotterdam Study cohort. ^b^Model 2: additionally adjusted for hypertension status, presence of dyslipidemia, daily alcohol consumption, daily physical activity, smoking status, highest attained level of education and serum glucose. ^c^Model 3: additionally adjusted for adherence to dietary guideline score.

**Supplemental Table 9. Associations between Ferric Reducing Ability of Plasma (FRAP) score and (probable) sarcopenia, stratified by age.**

|  |  | **Model 1^a^** | **p-value** | **Model 2^b^** | **p-value** | **Model 3^c^** | **p-value** |
| --- | --- | --- | --- | --- | --- | --- | --- |
| Probable sarcopenia  (n cases = 314)  *P for interaction 0.362* | Age <= 75.7  (n cases = 78) | 1.00  (0.76 ; 1.30) | 0.981 | 1.03  (0.79 ; 1.35) | 0.814 | 1.03  (0.78 ; 1.35) | 0.836 |
|  | Age > 75.7  (n cases = 236) | 0.90  (0.74 ; 1.09) | 0.289 | 0.93  (0.76 ; 1.13) | 0.446 | 0.94  (0.77 ; 1.15) | 0.544 |
| Sarcopenia  (n cases = 104)  *P for interaction 0.811* | Age <= 75.7  (n cases = 34) | 0.70  (0.46 ; 1.06) | 0.090 | 0.69  (0.45 ; 1.07) | 0.096 | 0.72  (0.47 ; 1.11) | 0.141 |
|  | Age > 75.7  (n cases = 70) | 0.80  (0.58 ; 1.11) | 0.191 | 0.87  (0.62 ; 1.22) | 0.414 | 0.87  (0.61 ; 1.22) | 0.414 |

N = 1001 participants aged <= 75.7 (sample median) years and N = 1000 participants aged > 75.7 years. Results are presented as odds ratios (OR) with corresponding 95% CI per 1 standard deviation increment in FRAP. ^a^Model 1: adjusted for sex, age and Rotterdam Study cohort. ^b^Model 2: additionally adjusted for hypertension status, presence of dyslipidemia, daily alcohol consumption, daily physical activity, smoking status, highest attained level of education and serum glucose. ^c^Model 3: additionally adjusted for adherence to dietary guideline score.

**Supplementary Figure 1. Overview of Rotterdam Study cohorts and measurement points.**

*Figure is included as a separate .tiff file.*
