# Supplementary figures and images for: Total Dietary Antioxidant Capacity and Longitudinal Trajectories of Body Composition
Source: Antioxidants (Basel). 2020 Aug 10;9(8):728. doi: 10.3390/antiox9080728 (PMC7465193; doi:10.3390/antiox9080728)

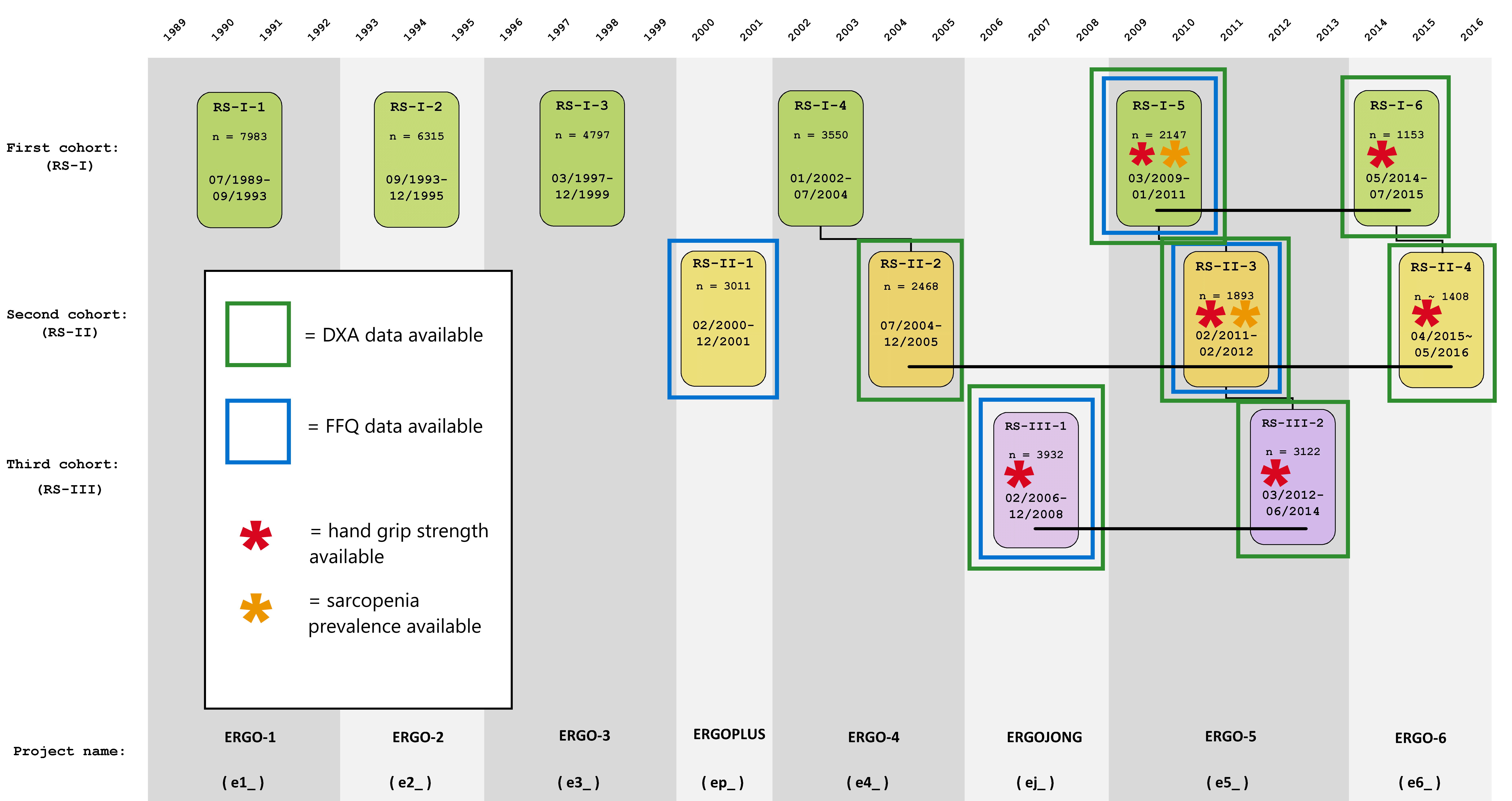

Supplement: Supplementary file 1 [file antioxidants-09-00728-s001.zip › supp_figure_vds.tiff]
